# Considerations when introducing MRI into a radiation therapy environment

**DOI:** 10.1002/jmrs.539

**Published:** 2021-08-25

**Authors:** Bronwyn Shirley, John Baines

**Affiliations:** ^1^ Radiation Therapy Townsville Cancer Centre Townsville Hospital and Health Service Townsville Queensland Australia

## Abstract

This issue of Journal of Medical Radiation Sciences includes two papers presenting different uses of magnetic resonance (MR) in radiation therapy (RT). With the advancement of MR‐simulators and Magnetic resonance linear accelerators (MRL), in addition to the use of diagnostic MR becoming more common place in the radiotherapy setting, there are a number of challenges to be considered. In this article, we present the perspectives of radiation therapists and medical physicists involved in the commissioning of an MRL in our centre. Image shows in‐house 3D printed supports mounted on the vendor‐supplied QA platform. The supports locate an array so that it is centred in the radiation field.

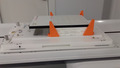

This issue of *Journal of Medical Radiation Sciences* includes two papers presenting different uses of magnetic resonance (MR) in radiation therapy (RT).^1^, ^2^ With the advancement of MR‐simulators and magnetic resonance linear accelerators (MRL), in addition to the use of diagnostic MR becoming more common place in the radiotherapy setting, there are a number of challenges to be considered. Recently, the Institute of Physics and Engineering in Medicine (IPEM) published guidelines for implementing MRI‐guided radiotherapy.^3^ Education, upskilling and collaboration are vital with the introduction of MR‐based technology. An appreciation of how diagnostic MR images are utilised in radiation therapy can also aid radiographers in understanding referrals, particularly patient positioning, scan length parameters and spatial resolution. In this editorial, we present the perspectives of radiation therapists and medical physicists involved in the commissioning of an MRL in our centre.

## MR Considerations in Radiation Therapy Planning

In investigating the clinical impact of MRI geometric distortion on gamma knife radiosurgery plans, Jacobsen et al produced a model that highlighted the severity of geometric image distortion, applying this to treatment plans to assess the clinical impact.^1^ They concluded that distortion leading to clinical impact is greater on smaller targets located at the image periphery. This highlights just one challenge that is present with the increased use of MR in RT. For this system and the MR scan protocol used, the authors measured the greatest effect 64.88 mm away from the isocentre and for smaller targets of 0.06cc. With the advances in diagnostic imaging, malignant lesions are now detected earlier, resulting in very small targets. Modifying a MR scan protocol to improve tumour visualisation or reduce scan time can affect the spatial accuracy of the image and this can result in a more drastic effect on the overall tumour target coverage leading to an undesirable clinical impact. Therefore, geometric distortion should be measured, and correction applied, for each scan protocol used in radiotherapy planning. Technology will evolve, and spatial accuracy and geometric distortion should be on the forefront of these advances in both software and hardware.

An MR scan in the planning position also requires the addition of MR safe patient immobilisation equipment as well as a hard top couch. Equipment needs to be assessed for both its interaction with the magnetic field, as well as interaction with the radiation and the patient within the magnetic field. The electron return effect (ERE) should be considered when there is a density change between the patient and equipment such as a vacbag. In such situations, the electron return effect can increase skin dose.^4^, ^5^ Spiralling contaminant electrons (SCE) and the electron streaming effect (ESE) can also increase unwanted skin dose to areas well outside of the patient, such as chin/face, ears and extremities. This should be considered in the planning stage to ensure that the patient is positioned in a way to reduce these effects and appropriate shielding such as bolus is considered to reduce unwanted skin dose. The patient also needs to be positioned in a way to reduce the chance of radiofrequency (RF) burns, for example, no skin‐to‐skin contact such as legs touching or holding hands.

The introduction of a planning MRI scan has the potential to change contouring for organs at risk, targets as well as planning risk volumes. Richardson et al. demonstrated a decrease in inter‐observer variability for urethra contouring by introducing a planning 3D T2 MRI.^2^ The logistics of obtaining a planning MRI scan may prove to be a burden on the patient or the health care system, however with departments now having access to their own MR Sim or MRL unit, these scans may soon be a regular part of the patient treatment pathway. This study compliments other studies around the world as many MRL departments investigate the feasibility of RO lite workflow for MRL treatment.^6^ Currently, a RO needs to be present for every MRL treatment to contour targets and approve the adapted radiation plan. Therefore, studies similar to this one could reduce the amount of time the RO is required to be present for a MRL treatment. This potential advance in scope of practice for a radiation therapist is very exciting; however, it still requires a great deal of research to ensure technical, ethical and legal standards are met.

## MR Considerations in Radiation Therapy Treatment

Prior to the clinical introduction of an MRL system, medical physicists will need to review current commercially available MR conditional equipment for the purposes of ongoing quality assurance (QA) requirements. The localisation options for quality assurance equipment set‐up on conventional linacs, such as field lights and lasers, are not available on MRL systems such as the Elekta Unity (Elekta AB, Stockholm, Sweden). In our department, the use of 3D printed frames for setting up phantoms, 2D arrays and ionisation chambers centrally located within the beam has facilitated time efficient procedures. In addition, the use of A‐P and L‐R electronic portal imaging device images provides a fast and reliable complementary verification of chamber and array positions for isocentric and isoplanar measurements, respectively. In our experience, vendor‐supplied phantoms for specific tasks, such as the determination of the coincidence of MR and linac MV isocentres will have dedicated localisation aids. Given the importance of MR image fusion with radiotherapy planning images in an adaptive planning workflow, an independent validation of isocentre coincidence is warranted. However, in the absence of commercially available equipment specific to this task, medical physicists currently need to develop in‐house methods. Elekta Unity adaptive planning algorithms, such as adapt to position and adapt to shape also confront medical physicists with the ‘how to verify the accuracy’ challenge. At this point in time, the need to develop standardised protocols and guidelines for quality assurance is evident. Processes and procedures to facilitate multisite intercomparisons and audits of the MRI and radiotherapy components of MRL systems need to be addressed.

Due to the magnetic field environment, there is potential out of field dose to the patient due to two sources as mentioned previously. Of the two sources of dose, the electron streaming effect (ESE) is the greater and has been associated with treatment beams entering and exiting the patient.^7^ However, it has been reported that ESE is also associated with treatment beams incident on the anterior MR imaging coil of the Elekta Unity.^8^ Consequently, use of the treatment planning system (TPS) that can calculate areas of ESE are necessary to determine appropriate positions for shielding. With current Unity beams bolus 1 cm thick is sufficient to attenuate ESE. We have found that measurements with EBT3 film provides a means to verify the ESE calculation accuracy of the treatment planning system.

Prior to and post‐magnet ramp up, X‐ray output and beam steering on a linac in a bunker adjacent to the Unity was investigated. Intercomparison of the output and steering servo systems demonstrated that for our facility, the static magnetic field of the Unity has a negligible influence on the linac. However, other Unity facilities would need to perform their own tests of the potential influence of the Unity on nearby conventional linacs. As part of medical physics commissioning, we verified the field strength at the 30 gauss location in the bunker and that the fringe field is less than 5 gauss in the console area. Noise levels within the bore of our MRL were also determined for MR sequences that would be used clinically. All our MRL patients treated wear both ear plugs and ear defenders. An ongoing audiology study for 20 fraction prostate treatments is being used to assess any potential hearing loss with prolonged exposure to MR noise.

Given the proximity of the linac beam generating components and the various coils of the imaging system in an MRL, the question of crossover effects arises. Given that MR images may be acquired during treatment beam delivery, it is important to verify the extent to which beam‐on effects MR images and MR sequences impact on dosimetry. Appropriate measurements should be performed as part of physics commissioning.

## Safety Considerations

The MRL bunker and surrounding areas at our facility were considered in terms of the zones (1–4) as defined by The Royal Australian and New Zealand College of Radiologists (RANZCR).^9^ Given our limited staff experience with MRI systems, a permanent magnet and handheld ferromagnetic/metal detector was acquired to assist with staff and patient screening prior to entry into zone 3, the maze at our facility. Timely sourcing an MR conditional patient trolley, fire extinguisher and trolley for physics QA equipment are needed. As part of best practice for the safe operation of the MR system, RANZCR‐type level 1 and level 2 training for staff is required with annual reviews.^9^ Prior to our go live date, an independent audit of our MR facility, policies and procedures, including emergency evacuation was performed by an external expert in the field.

## Training and Upskilling

There is a need for upskilling of radiation therapists and radiation oncology medical physicist to be able to safely operate and preform QA on an MRI machine as well as to thrive in potential research opportunities. At present, there is a limited number of pathways for a radiation therapist and radiation oncology medical physicist to receive formal and accredited MR in RT training within Australia. There are a number of short courses, however, these are not yet tailored for this profession and are not adequate to fully learn and understand MRI technology. There are a few postgraduate courses available which could prove to be very beneficial; however, these courses are a heavy investment for the individual and also include course material that is not relevant to a radiation therapy environment. There is a strong need for an MRI accredited program that is to be directed towards the radiation therapy setting with hands on training. Clinical placement may prove to be difficult to achieve due to the limited number of centres within Australia that utilise this technology.

## Multidisciplinary Team Approach

A multidisciplinary team approach when implementing a new form of technology into daily standard practice need to be considered. One of the most valuable resources that a radiation therapy department may have access to when considered MR implementation is the medical imaging diagnostic department. When implementing the MRL at Townsville Cancer Centre (TCC), the collaboration with the medical imaging department was invaluable. From setting up safety protocols to onsite MR experience and image interpretation, they were key to the success of many aspects of the MRL program at TCC and will continue to be instrumental in future developments. Other departments may have opportunity to employ a MR radiographer. This has been beneficial as it allows for cross‐disciplinary training.^10^ IPEM guidelines mentioned previously also highlight the importance of a multidisciplinary team as well as multi‐institutions approach when putting forward guidelines.^3^

## Conclusion

While there are challenges in incorporating MR into the radiation therapy environment as briefly outlined in this editorial, there are many advantages in improving patient care. Just like any other technology that has been introduced into the radiation therapy department in the past, an extensive multidisciplinary team approach is warranted to guarantee for the success of the MR implementation. There is a gap within market for MR safe equipment that can be used for QA, as well as formal education for both radiation oncology medical physicist and radiation therapist. MR in RT is getting a lot of attention in the literature and therefore these gaps are on the way to being resolved.

## Conflict of Interest

The authors declare no conflict of interest.
